# Calculation of Mechanical Properties, Electronic Structure and Optical Properties of CsPbX_3_ (X = F, Cl, Br, I)

**DOI:** 10.3390/molecules28227643

**Published:** 2023-11-17

**Authors:** Yang Liu, Canxiang Fang, Shihe Lin, Gaihui Liu, Bohang Zhang, Huihui Shi, Nan Dong, Nengxun Yang, Fuchun Zhang, Xiang Guo, Xinghui Liu

**Affiliations:** 1 School of Physics and Electronic Information, Yan’an University, Yan’an 716000, Chinayadxynx@1236.com (N.Y.); 2 Science and Technology on Aerospace Chemical Power Laboratory, Hubei Institute of Aerospace Chemotechnology, Xiangyang 441003, Chinaguoxiang@casc42.cn (X.G.)

**Keywords:** first principles, CsPbX_3_ (X = F; Cl; Br; I), mechanical properties, electronic structure, optical properties

## Abstract

We utilized a first-principle density functional theory for a comprehensive analysis of CsPbX_3_ (X = F, Cl, Br, I) to explore its physical and chemical properties, including its mechanical behavior, electronic structure and optical properties. Calculations show that all four materials have good stability, modulus of elasticity, hardness and wear resistance. Additionally, CsPbX_3_ demonstrates a vertical electron leap and serves as a semiconductor material with direct band gaps of 3.600 eV, 3.111 eV, 2.538 eV and 2.085 eV. In examining its optical properties, we observed that the real and imaginary components of the dielectric function exhibit peaks within the low-energy range. Furthermore, the dielectric function gradually decreases as the photon energy increases. The absorption spectrum reveals that the CsPbX_3_ material exhibits the highest UV light absorption, and as X changes (with the increase in atomic radius within the halogen group of elements), the light absorption undergoes a red shift, becoming stronger and enhancing light utilization. These properties underscore the material’s potential for application in microelectronic and optoelectronic device production. Moreover, they provide a theoretical reference for future investigations into CsPbX_3_ materials.

## 1. Introduction

In 1839, Gustav Rose discovered the mineral CaTiO_3_ and named it perovskite [1]. Perovskite is a class of mineral compounds characterized by the general formula ABO_3_ [2]. Halide perovskites possess a similar crystal structure characterized by the general formula ABX_3_. Organic–inorganic halide chalcogenides combine the solution-processable characteristics of organic materials and the excellent optoelectronic properties of inorganic materials with excellent photophysical properties, such as high absorption coefficients, long exciton diffusion distances, high carrier mobility, low exciton binding energies, etc., and the photovoltaic devices constructed with them have a simple preparation process, inexpensive production costs, excellent flexibility and outstanding optoelectronic performance [3]. Their structure typically consists of octahedral coordination, where A and B represent two different-sized cations (usually A = Rb^+^ or Cs^+^, B = Sn^+^ or Pb^+^) and X represents an anion (halides: X = F^−^, Cl^−^, Br^−^ and I^−^) [4]. CsPbX_3_ (X = Cl, Br, I) is a chalcogenide optoelectronic material consisting of Cs^+^ and Pb^+^ ions and X^−^ ions (Cl^−^, Br^−^, I^−^), focusing on its electronic structure and luminescence properties [5].

In the face of the rapidly increasing energy demand, the focus on saving energy and environmental issues [6], photocatalysis has become an essential strategy for solving the problems of carbon emissions and energy supply simultaneously by using solar energy [7]. Halide lead perovskite materials have gained significant attention in photocatalysis due to their wide, tunable light absorption range, low cost and abundant surface-active sites [8]. They have shown a good photocatalytic performance in CO_2_ reduction [9] and aquatic hydrogen [10]. For instance, in 2016, Nam et al. first reported the application of metal halide perovskite materials as catalysts in photocatalysis research [11]. The practical application of organic–inorganic hybrid perovskite materials (such as MAPbX_3_ or FAPbX_3_) is limited due to their structural instability, which arises from the volatile nature of the organic cations. These materials are prone to irreversible decomposition in polar solvents, forming PbX_2_ (X = I, Br, Cl) precipitates, organic cations (MA/FA) and halide anions. It has been shown that the photothermal stability of the material can be significantly improved if an all-inorganic chemical structure is adopted, such as when an organic cation at the A^−^ site is replaced by inorganic Cs^2+^ [12]. A higher photoluminescence quantum yield, excellent stability and narrow-band emission properties are achieved compared to the organic–inorganic hybrid analogues. Inorganic CsPbX_3_ (X = Cl, Br, I) perovskite materials, being characterized by high photoluminescence efficiency (>90%), a broad absorption range, long electron–hole diffusion length and stable crystal structure, have garnered significant attention in the field of photocatalysis [13,14,15,16]. Sebastian et al. successfully synthesized CsPbBr_3_ and CsPbCl_3_ perovskite crystals and analyzed their band gaps through photoluminescence spectroscopy [17]. Heidrich et al. [18,19] carried out extensive studies on the electronic structure and spectra of CsPbCl_3_ and CsPbBr_3_ and determined that CsPbI_3_, CsPbBr_3_, CsPbCl_3_ and CsPbF_3_ have lattice constant values of 6.2894 Å, 5.874 Å, 5.605 Å and 4.7748 Å, respectively [20,21,22]. Murtaza and Ahmed used the FP-LAPW method to analyze the structural [23], electronic and optical properties of CsPbM_3_ (M = Cl, Br, I) and found that the lattice constant increases as the halide ion transitions from Cl to I. The calculated band gap values were 2.55 eV, 2.19 eV and 1.75 eV for CsPbF_3_, CsPbCl_3_ and CsPbBr_3_, respectively [24]. However, the vast majority of these studies focused on one aspect only and neglected comprehensive analysis, thus lacking a comprehensive understanding of the properties of CsPbX_3_ (X = F, Cl, Br, I) and in-depth investigation and comprehensive comparison.

Our current work aims to systematically investigate the mechanical properties, electronic structure and optical properties of CsPbX_3_ (X = Cl, Br, I) based on first-principle calculations, providing a theoretical reference for further studies on CsPbX_3_ materials in the future.

## 2. Results and Discussion

### 2.1. Mechanical Properties

In this paper, the elastic constant tensor C_ij_ is calculated using the stress–strain method, and the bulk modulus of elasticity (B), Young’s modulus (E), shear modulus (G), Poisson’s ratio (ν) and hardness (H) are calculated with the Voigt–Reuss–Hill (VRH) approximation [25,26]. The calculated equations are as follows (Equations (1)–(11)). Hardness is estimated using Tian’s model [27], and elastic constants are solved via the stress–strain method. The elastic constant is an important parameter in the study of the mechanical and dynamic characteristics of materials and can be used to estimate the hardness and stability of materials.

The mechanical stability criterion is used to determine whether the system satisfies the elastic stability mechanism, and is as follows:

C_ij_ > 0 (1)

C_ii_ + C_jj_ − 2C_ij_ > 0 (2)

C_11_ + C_22_ + C_33_ + 2(C_12_ + C_13_ + C_23_ ) > 0 (3)

Table 1 lists the elastic constants of the four systems’ C_ij_. The stability criterion confirms that all three of these systems, CsPbF_3_, CsPbCl_3_, CsPbBr_3_ and CsPbI_3_, satisfy the mechanical stability conditions for orthorhombic crystal structures. And the small difference between the calculated and actual values proves the results’ reliability.

The formula for calculating the mechanical properties is as follows:

(4) $$B_{V}=\frac{\left(C_{11}{+2C}_{12}{+2C}_{13}{+C}_{22}{+2C}_{23}{+C}_{33}\right)}{9}$$

(5) $$B_{R}=\frac{1}{{(C}_{11}{+C}_{22}C_{33}{)+2(C}_{12}{+C}_{13}C_{23})}$$

(6) $$G_{V}=\frac{C_{11}-C_{12}-C_{13}+C_{22}-C_{23}+C_{33}+3C_{44}+3C_{44}+3C_{55}+3C_{66}}{15}$$

(7) $$G_{R}=\frac{15}{{4(C}_{11}{+C}_{22}{+C}_{33})-{4(C}_{12}{+C}_{13}{+C}_{23}{)+3(C}_{44}{+C}_{55}{+C}_{66})}$$

The bulk modulus of elasticity (*B*), Young’s modulus (*E*), shear modulus (*G*) and Poisson’s ratio (*ν*) can be calculated as follows:

(8) $$B=\frac{1}{2(B_{R}+B_{V})}$$

(9) $$G=\frac{1}{2(G_{R}+G_{V})}$$

(10) $$E=\frac{9BG}{3B+G}$$

(11) $$\nu =\frac{3B-2G}{2(3B+G)}$$

**Figure 1:**
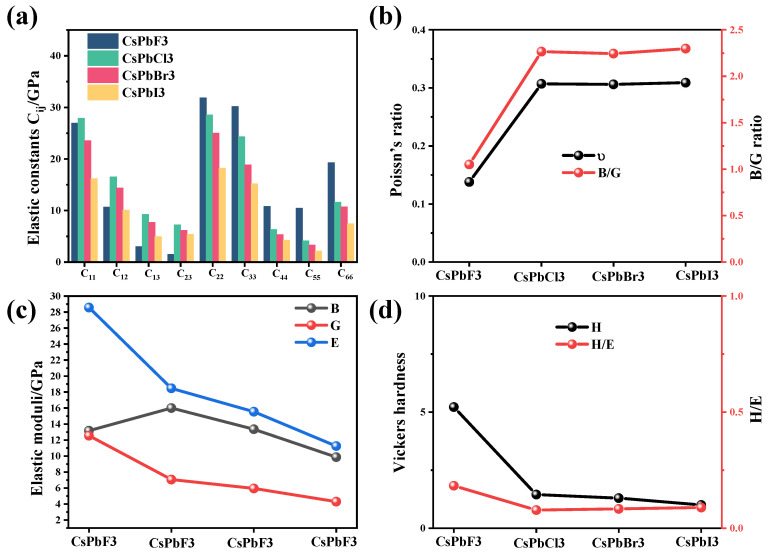
(**a**) Elastic constants, (**b**) Poisson’s ratio and B/G, (**c**) modulus of elasticity and (**d**) hardness and H/E for CsPbX_3_ (X = Cl, Br, I). Bulk modulus (B), shear modulus (G), Young’s modulus (E), Poisson’s ratio (ν) and hardness (H).

The bulk modulus is a parameter that reflects the ability of a material to resist volume change. Generally speaking, stiff materials have a high bulk modulus. To show the variation in the elastic constants more clearly, the results are plotted in Figure 1a, from which it can be seen that most of the elastic constants decrease as the atomic radius of element X increases.

Table 2 lists the bulk modulus (*B*), shear modulus (*G*), Young’s modulus (*E*), Poisson’s ratio (*ν*) and hardness (*H*) of the four materials. The effects of different elements on the above results can be compared more clearly in Figure 1b–d.

Figure 1c shows that the four elastic moduli show a trend of increasing and then decreasing as the halogenated elements’ radius increases from large to small with the change in X elements. The bulk modulus and shear modulus indicate the material’s resistance to volume and shape changes, respectively, and the larger the value, the stronger the resistance. The bulk modulus describes the elasticity of a homogeneous isotropic solid and can be expressed as a force per unit area indicating incompressibility. However, from the calculated elastic constants, it can be seen that the C_12_, C_13_ and C_23_ of the elastic constants of CsPbF_3_ are very small, so its bulk modulus is the smallest among all three materials.

When the bulk modulus exceeds the shear modulus, the material changes its shape rather than its volume when subjected to external forces. Young’s modulus is the longitudinal modulus of elasticity, which characterizes the resistance of the material to deformation, and the larger the value of Young’s modulus, the greater the stiffness of the material. From the results, it can be determined that CsPbF_3_, CsPbCl_3_, CsPbBr_3_ and CsPbI_3_ are hard materials.

Based on Pugh’s [28] empirical relationship, the ratio of the bulk modulus B to the shear modulus G can determine the tough–brittle behavior of the material. When B/G > 1.75, the material is ductile, and vice versa. Poisson’s ratio (ν) reflects the stability of the crystal against shear and can also be used to determine the intrinsic toughness of the material; if ν is greater than 0.26, the material exhibits relative toughness, and if not, the material is brittle. It is shown in Figure 1b that all three materials except CsPbF_3_ exhibit relative ductility, while CsPbF_3_ is brittle.

The hardness of a material characterizes, to some extent, the ability of the material to resist deformation or fracture, and according to the theory of Tian et al. [27] the hardness of a material can be derived from formulae such as the shear modulus, which is calculated as

H = 0.92 K^1.137^ G^0.708^ (12)

K = G/B(13)

Wear resistance is an essential indicator for materials in extreme environments. According to the criteria proposed by Leyland and Matthews, the elastic breaking strain H/E can be used to characterize the wear resistance of an alloy. Figure 1d shows the hardness values and H/E of the four materials, from which it can be seen that CsPbF_3_ has the highest Vickers hardness and wear resistance, while the other three materials are similar.

### 2.2. Electronic Properties

As shown in Table 3, the band gap was evaluated using the PBE and SCAN function for the various CsPbX_3_ structures. PBE underestimates the band gap compared to the experimental data, so we used SCAN to perform a new calculation of the band gap of the material. Although the band gap obtained from PBE calculations underestimates the band gap, the other results obtained from PBE calculations are reliable [29].

But these two general functions produce almost identical trends in the calculated band gaps for the four structures, i.e., CsPbF_3_ > CsPbCl_3_ > CsPbBr_3_ > CsPbI_3_, even though their predictions are quite different from each other.

**Figure 2:**
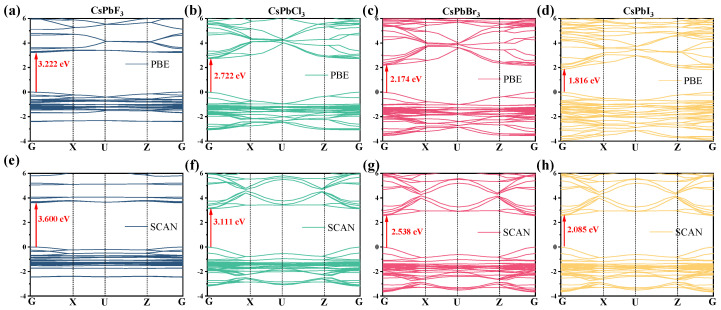
(**a**–**d**) are the electronic energy band structures calculated using the PBE method, and (**e**–**h**) are the electronic energy band structures calculated using the SCAN method.

Figure 2 shows the energy band structure of CsPbX_3_. The band gaps of CsPbF_3_, CsPbCl_3_, CsPbBr_3_ and CsPbI_3_ obtained using the PBE method are 3.222 eV, 2.722 eV, 2.174 eV and 1.816 eV, respectively (Figure 2a–d), while the band gaps calculated using the SCAN method are 3.600 eV, 3.111 eV, 2.538 eV and 2.085 eV, respectively (Figure 2e–h). Furthermore, as can be seen in Figure 2, the minimum value of the conduction band (CB) and the maximum value of the valence band (VB) in all four structures are obtained near the G point, which characterizes the band gap structure as a direct band gap.

At the same time, introducing different halogen atoms leads to a gradual downward shift of the CBM, leading to a smaller forbidden bandwidth. The band gap width values are close to the experimental values. The formation of a direct band gap and the small forbidden bandwidth facilitate the efficient use of visible light. The lifetime of carriers in direct band gap semiconductors tends to be very short; at the same time, this direct composite can put out almost all of the energy in the form of light, making the material highly efficient in luminescence.

**Figure 3:**
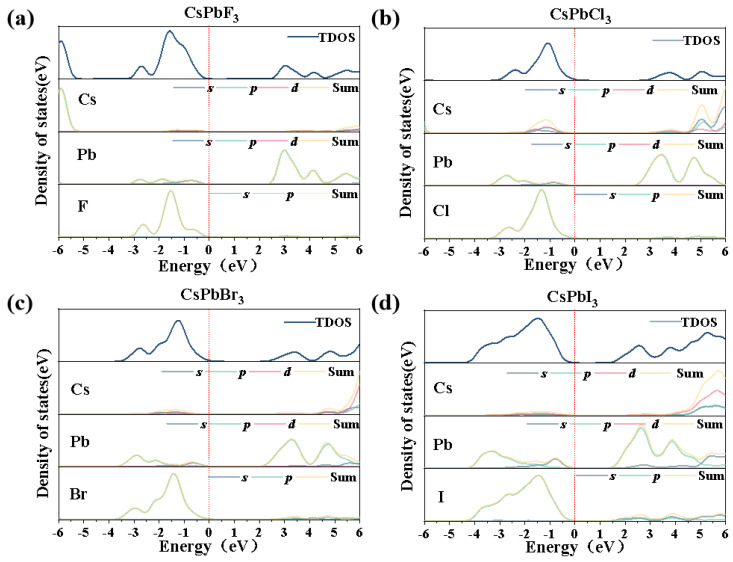
Densities of states and fractional-wave densities of states for (**a**) CsPbF_3_, (**b**) CsPbCl_3_, (**c**) CsPbBr_3_, (**d**) CsPbI_3_.

Figure 3 shows the density of states for the four catalysts. As can be seen from the figure, for the four CsPbX_3_ semiconductors, the conduction band is mainly composed of Pb and Cs, while the valence band is mainly occupied by halogen elements (F, Cl, Br, I), but exhibits different forbidden bandwidths due to the halogens, which vary in relation to the dispersion of the halogens at the Fermi energy level near the conduction band; the lower the dispersion, the smaller the forbidden bandwidth, leading to different excitation capabilities of the photogenerated electron–hole pairs.

### 2.3. Optical Properties

The optical properties of the four materials are calculated by applying first-principal simulation. The frequency-dependent dielectric function characterizes the linear response of a solid to electromagnetic radiation and consists of two components: the real part ε_1_(ω) and the imaginary part ε_2_(ω). These quantities can be determined by analyzing the jump matrix and the dielectric function relationship [34].

The dielectric function can be expressed as the following equation [35,36]:

(14) $$\varepsilon \left(\omega \right)=\varepsilon_{1}\left(\omega \right)+i\varepsilon_{2}\left(\omega \right)$$

The equation of ε_2_(ω) is expressed as follows [37]:

(15) $$\varepsilon_{2}\left(\omega \right)=\frac{8\pi^{2}e^{2}}{\omega^{2}m^{2}}\sum_{n}\sum_{n^{\prime }}\int_{BZ}{\left|P_{nn^{\prime }}^{\nu }\left(k\right)\right|}^{2}f_{kn}\left(1-f_{kn^{\prime }}\right)\partial \left(E_{n}^{k}-E_{n^{\prime }}^{k}-h\omega \right)\frac{\partial^{3}k}{2\pi^{3}}$$

Here,$f_{kn}$, $\left.P_{nn^{\prime }}^{\nu }\left(k\right)\right.$ is the projection in the direction ν of the electric field about the momentum dipole–matrix element, $E_{n}^{k}$(k) represents the energy of a lone electron, and e and m are the charge and mass of one electron, respectively.

The equation of ε_1_(ω) is expressed as follows [38]:

(16) $$\varepsilon_{1}\left(\omega \right)=1+\frac{2}{\pi }P\int_{0}^{\infty }\frac{\omega^{\prime }\varepsilon_{2}\left(\omega^{\prime }\right)}{\omega^{\prime 2}-\omega^{2}}d\omega^{\prime }$$

The discussion on the dielectric function ε(ω) leads to some important optical properties. We can calculate the absorption coefficient α(ω), refractive index n(ω), reflectivity R(ω), conductivity σ(ω) and energy loss functions L(ω) from ε_1_ (ω) and ε_2_ (ω). The specific formulae are as follows [39,40,41]:

(17) $$\alpha (\omega )=\sqrt{2}{\left[\sqrt{\varepsilon_{1}^{2}(\omega )+\varepsilon_{2}^{2}(\omega )}-\varepsilon_{1}(\omega )\right]}^{\frac{1}{2}}$$

(18) $$n(\omega )=\frac{1}{\sqrt{2}}{\left[\sqrt{\varepsilon_{1}^{2}(\omega )+\varepsilon_{2}^{2}(\omega )}+\varepsilon_{1}(\omega )\right]}^{\frac{1}{2}}$$

(19) $$R(\omega )=\frac{{\left[n(\omega )-1\right]}^{2}+k^{2}(\omega )}{{\left[n(\omega )+1\right]}^{2}+k^{2}(\omega )}$$

(20) $$\sigma (\omega )=\frac{\omega }{4\pi }\varepsilon_{2}(\omega )$$

(21) $$L(\omega )=\frac{\varepsilon_{2}(\omega )}{\varepsilon_{1}^{2}(\omega )+\varepsilon_{2}^{2}(\omega )}$$

**Figure 4:**
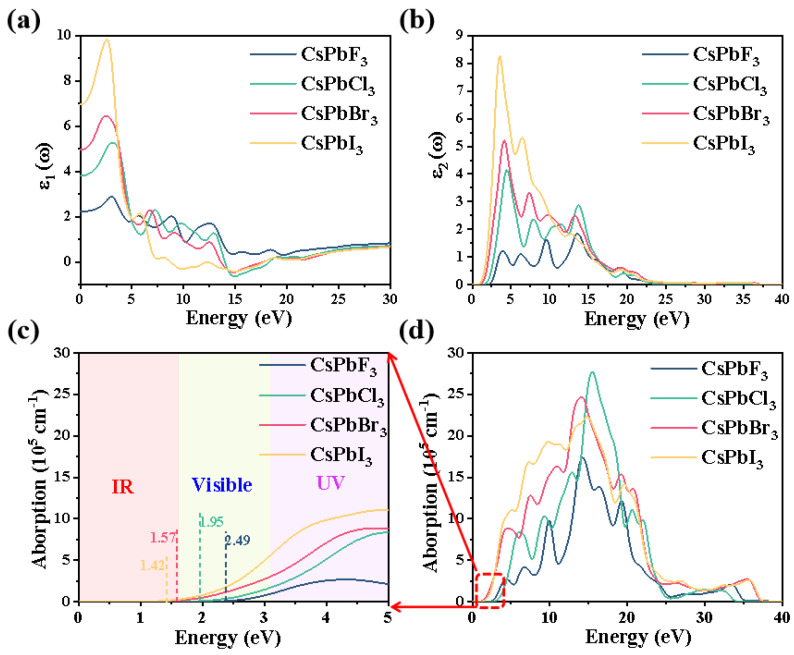
(**a**) Real and (**b**) imaginary parts of the dielectric function and (**c**,**d**) light absorption intensity (local amplification) of CsPbX_3_ (X = F, Cl, Br, I).

The real part ε_1_ and imaginary part ε_2_ curves of the dielectric function of CsPbX_3_ with energy are shown in Figure 4a,b. The imaginary part of the dielectric function is related to the electronic transition, so the imaginary part of the dielectric function can reflect the strength of the electron-stimulated transition and thus indicate the strength of the optical absorption capacity. The peaks of ε_1_ and ε_2_ are distributed in the low-energy region (≤15 eV). In the high-energy region (>15 eV), ε_1_ tends to 1 and ε_2_ tends to 0. Currently, CsPbX_3_ absorbs very few incident photons and has a high transmittance. The values on the vertical axis at zero energy are the static dielectric constants of the materials, which are 2.22, 3.81, 4.97 and 6.95 for CsPbF_3_, CsPbCl_3_, CsPbBr_3_ and CsPbI_3_, respectively (Figure 4a), showing a gradual increase in the static dielectric constant. With the replacement of different halogen elements, the static permittivity function gradually increases, which is due to the band gap value gradually decreasing. Furthermore, electrons require very little energy to jump from the top of the valence band to the bottom of the conduction band and are easily polarized.

Figure 4b shows the imaginary part of the dielectric function of the CsPbX_3_ system. The imaginary part of the dielectric function indicates the intensity of electron transitions, thereby reflecting the absorption capacity of light. There are four dielectric peaks between 0 and 15 eV for CsPbF_3_, CsPbCl_3_, CsPbBr_3_ and CsPbI_3_, respectively. As the band gap decreases, the photon energy required for the electron leap decreases, so the dielectric peaks move towards the lower-energy region and the peaks in the dielectric imaginary part move towards the lower-energy region.

Figure 4c,d show the absorption coefficients of the CsPbX_3_ system. The absorption edge of the system gradually extends into the infrared band; i.e., the absorption spectra are all significantly red-shifted, and the magnitude of the edge values follows the order CsPbI_3_ < CsPbBr_3_ < CsPbCl_3_ < CsPbF_3_, increasing the absorption range of the system in the infrared. It can be seen from the graph that the absorption coefficient of the system increases with the transformation of the halogen atoms with larger ionic radii, and CsPbI_3_ has the largest red shift and the strongest light absorption intensity. This shows that by changing the halogen atoms in the CsPbX_3_ system, it is possible to enhance its light absorption in the infrared region, which provides a theoretical basis for the application of the system in optics.

**Figure 5:**
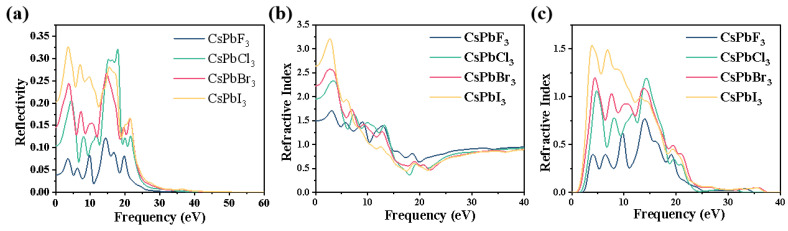
(**a**) Reflection and (**b**,**c**) refraction spectra of CsPbX_3_ (X = F, Cl, Br, I).

Figure 5a shows the reflectance spectrum of the CsPbX_3_ system, reflecting the trend of the reflectance as the photon energy changes. The reflectance of CsPbF_3_, CsPbCl_3_ and CsPbBr_3_ has a maximum peak at photon energies of 14.391 eV, 17.852 eV and 14.637 eV, and the reflectance of CsPbCl_3_ and CsPbBr_3_ is greater than 0.25, but the reflectance of CsPbF_3_ is smaller and less than 0.15. The reflectance of both CsPbCl_3_ and CsPbBr_3_ is less than 0.25 at 13.844 eV. Although the trend of the increasing reflectance of CsPbI_3_ remains the same, its reflectance is at a maximum energy of 3.638 eV. The reflectance of the CsPbX_3_ system is less than 0.33, so a small portion of the incident light is reflected back. The reflectivity tends to zero as the photon energy increases above the 21.614 eV range.

Meanwhile Figure 5 shows the curves of the refractive index and extinction coefficient with photon energy, as shown in Figure 5b. The static refractive indices n(0) of CsPbF_3_, CsPbCl_3_, CsPbBr_3_ and CsPbI_3_ are 1.49, 1.95, 2.22 and 2.63, respectively, and the static refractive index of CsPbI_3_ is the largest. The corresponding energies are 3.16 eV, 3.41 eV, 2.90 eV and 2.79 eV. Between 3.5 eV and 19 eV, the refractive index of the whole system tends to decrease with increasing photon energy, and the curves almost coincide after 34 eV, after which the refractive coefficient of the system remains almost constant around 0.93. Figure 5c shows the extinction coefficient curve, which has five peaks, decreasing towards zero after 24.14 eV, and remaining constant at almost zero after reaching 37.43 eV. The overall trend of the curve in Figure 5c shows that the curve tends to increase as the ionic radius of the replacement halogen atoms becomes larger, indicating that the change in the system increases the extinction coefficient, indicating that the energy loss increases as the light waves propagate through the absorbing medium, weakening the utilization of light by the material.

**Figure 6:**
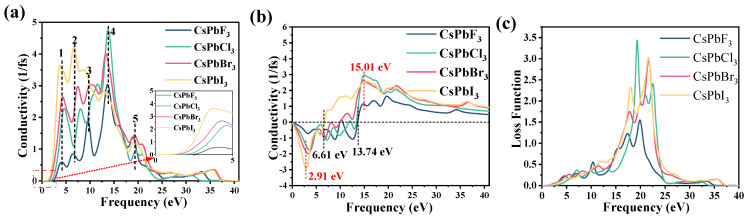
(**a**,**b**) Conductivity and (**c**) loss functions of CsPbX_3_ (X = F, Cl, Br, I).

Photoconductivity is an important parameter in the study of optoelectronic materials. It describes the phenomenon of light-induced changes in the conductivity of semiconductors. Figure 6a,b show the photoconductivity of CsPbX_3_, with a exhibiting the real part of the photoconductivity and b exhibiting the imaginary part of the photoconductivity. Photoconductivity is an important parameter in the study of optoelectronic materials and describes the phenomenon of light-induced changes in the electrical conductivity of a semiconductor. As shown in Figure 6a, the starting energy of the photoconductivity corresponds to the same trend as the change in the band gap size ratio shown in the previous energy band structure. As the atomic radius of element X becomes larger, the conductivity peak moves towards the bottom energy region, with five conductivity peaks appearing in all systems. The peaks of the other three systems (CsPbCl_3_, CsPbBr_3_ and CsPbI_3_) increase with an increasing atomic radius of X. These changes are caused by changes in the energy band structure and are consistent with the pattern of changes in the imaginary part of the dielectric function. Furthermore, the peak of the imaginary part of the photoconductivity is located along the falling edge of the real part of the photoconductivity, and the valley of the imaginary part of the photoconductivity occurs along the rising edge of the real part of the photoconductivity, with its peak near the positions of 2.91 eV and 15.04 eV (Figure 6b).

Figure 6c shows the energy loss function obtained from the analysis of the theoretical calculations. The calculated results show that the maximum energy loss peaks for CsPbF_3_, CsPbCl_3_, CsPbBr_3_ and CsPbI_3_ are at 19.85 eV, 19.33 eV, 21.73 eV and 21.68 eV, respectively, and the energy loss function peaks at 1.56 eV, 3.44 eV, 2.96 eV and 3.05 eV thereafter. After that, the energy loss function spectrum drops sharply to zero. The energy loss function of CsPbX_3_ shows a sharp increase and then a decrease in the range of 10–25 eV for the photoelectron energy but tends to zero in all other ranges, indicating that there is no excessive energy loss in these ranges.

## 3. Computation Method

CsPbX_3_ belongs to the Pbnm space group, and its detailed structural parameters are shown in Table 3 below. The optimized crystallographic information file of the four structures of CsPbX_3_ is provided in the supplementary information. A method based on the generalized gradient approximation (GGA) method proposed by Perdew–Burke–Ernzerhof (PBE) based on first-principle density functional theory is used to solve the exchange–correlation energy generalization [42], which is implemented in the CASTEP code [43]. Geometric optimization is performed using the limited memory Broyden–Fletcher–Goldfarb–Shanno (LBFGS) method [44], and core electron interactions are calculated using an ultrasoft pseudopotential [45]. A 2 × 2 × 1 k-point and an energy cut-off of 400 eV were used for geometrical optimization and property calculations. In addition, the energy convergence tolerance during atomic relaxation was set to no more than 1 × 10^−5^ eV/atom, and the atomic force was limited to less than 0.03 eV/Å with a maximum displacement of 0.001 Å.

Considering that the PBE general function may be underestimated, we use the Strongly Constrained and Appropriately Normed Semilocal Density Functional (SCAN) general function to calculate the band gap accurately [46]. Systematic tests of SCAN generalization have shown that this kind of generalization provides a significant improvement over GGA in the calculation of various properties (especially energy-dependent properties) of various solids, almost to the level of heterogeneous generalization, but saves a significant amount of time as compared to when heterogeneous generalization is used, keeping the computational effort at the level of semilocal generalization.

## 4. Conclusions

In this paper, the electronic structure and optical properties of CsPbX_3_ are calculated and analyzed using a first-principle plane wave pseudopotential approach in the framework of density generalized theory. The results show that CsPbF_3_, CsPbCl_3_, CsPbBr_3_ and CsPbI_3_ are all semiconductor materials with direct band gaps of 3.600 eV, 3.111 eV, 2.538 eV and 2.085 eV, which are close to the experimental values. The bottom of the conduction band is dominated by contributions from Pb and Cs, and the top of the valence band is dominated by contributions from halogenated states. Optical analysis shows that the peaks of both the real and imaginary parts of their dielectric functions occur in the low-energy region and that the dielectric function decreases slowly as the photon energy increases. The absorption spectrum of light shows that the CsPbX_3_ material absorbs the most UV light, and that with an increase in the radius of the X atom, the light absorption shows a red shift and the light absorption becomes stronger, i.e., the utilization of light increases. These properties establish the usefulness of the material for the fabrication of microelectronic and optoelectronic devices and provide a potential application and a theoretical reference for further research on CsPbX_3_ materials. 

## Figures and Tables

**Table 1:** Elastic constants of materials (unit: Gpa).

System	C_11_	C_12_	C_13_	C_23_	C_22_	C_33_	C_44_	C_55_	C_66_
CsPbF_3_	26.944	10.646	3.018	1.472	31.857	30.189	10.787	10.463	19.287
CsPbCl_3_	27.868	16.536	9.232	7.208	28.541	24.298	6.298	4.122	11.598
CsPbBr_3_	23.542	14.357	7.679	6.120	24.981	18.857	5.313	3.298	10.711
CsPbI_3_	16.193	10.061	4.949	5.355	18.191	15.175	4.266	2.126	7.425

**Table 2:** Mechanical properties of the material: bulk modulus (GPa), shear modulus (GPa), Young’s modulus (GPa), Poisson’s ratio(GPa) and hardness(GPa).

System	Bulk Modulus (B)	Shear Modulus (G)	Young’s Modulus (E)	Poisson’s Ratio (ν)	Hardness (H)	B/G	H/E
CsPbF_3_	13.170	12.542	28.560	0.138	5.215	1.050	0.183
CsPbCl_3_	16.008	7.064	18.474	0.307	1.448	2.266	0.078
CsPbBr_3_	13.361	5.954	15.553	0.306	1.298	2.244	0.083
CsPbI_3_	9.870	4.295	11.253	0.309	1.002	2.298	0.089

**Table 3:** Crystal structure information and band gap of CsPbX_3_ (X = F, Cl, Br, I). I and II in the Pb-X and Pb-X-Pb columns of the table represent the different lengths, and different angles, of the bonds formed by the Pb atoms with the X atoms (specific types can be viewed in the Supplementary Material for picture information).

System	Lattice Constant/Å			Unit Cell Volume V/Å^3^	Band Gap/eV			Pb-X Bond Length/Å		Pb-X-Pb Bond Angle/°	
	a	b	c		PBE	SCAN	Exp.	I	II	I	II
CsPbF_3_	6.950	7.030	10.095	493.149	3.222	3.600	3.68 [30]	2.494/2.493	2.550	164.758	163.341
CsPbCl_3_	8.030	8.112	11.389	741.918	2.722	3.111	2.90 [31]	2.918/2.915	2.951	156.167	149.494
CsPbBr_3_	8.388	8.504	11.869	846.726	2.174	2.538	2.30 [32]	3.060/3.056	3.097	155.108	146.726
CsPbI_3_	8.894	9.087	12.587	1017.39	1.816	2.085	~1.70 [33]	3.273/3.271	3.307	152.672	144.151

## Data Availability

The authors are willing to provide the supporting data of this study upon reasonable request.

## Supplementary Materials

The following supporting information can be downloaded at: https://www.mdpi.com/article/10.3390/molecules28227643/s1. Figure S1: The cell structure of CsPbX_3_. Figure S2: Details of the four structures optimized (CIF). Figure S3: The different bond lengths and bond angles in the four structures. Figure S4: CASTEP Geometry Optimization and Optimization Convergence.
