# Supplementary material for: Calculation of Mechanical Properties, Electronic Structure and Optical Properties of CsPbX3 (X = F, Cl, Br, I)
Source: Molecules. 2023 Nov 17;28(22):7643. doi: 10.3390/molecules28227643 (PMC10673478; doi:10.3390/molecules28227643)

Supplementary Information

Figure S1. The cell structure of CsPbX3

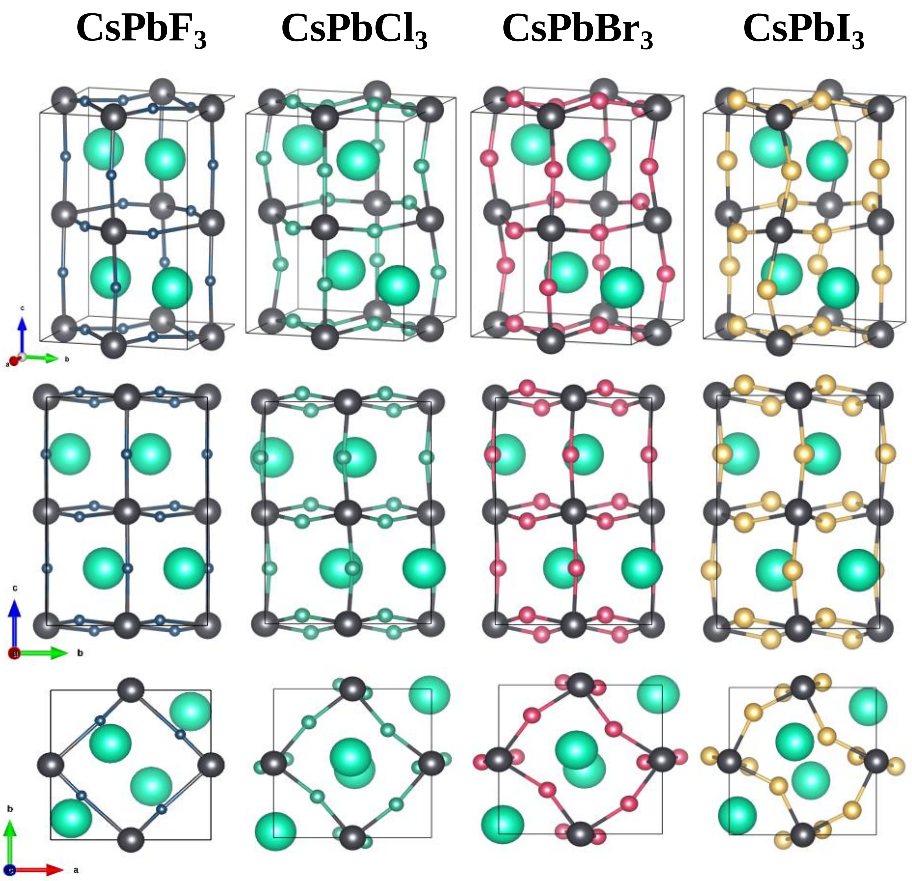

Figure S2. Details of the four structures optimized (CIF)

```
CsPbF3
1.0
        8.2069997787         0.0000000000         0.0000000000
        0.0000000000         8.2550001144         0.0000000000
        0.0000000000         0.0000000000        11.7589998245
    F    Cs    Pb
   12     4     4
Direct
     0.691559970         0.203950003         0.023830000
     0.308440030         0.796050012         0.976170003
     0.191559970         0.296050012         0.976170003
     0.808440030         0.703949988         0.023830000
     0.308440030         0.796050012         0.523829997
     0.691559970         0.203950003         0.476170003
     0.808440030         0.703949988         0.476170003
     0.191559970         0.296050012         0.523829997
     0.963020027         0.503239989         0.250000000
     0.036979999         0.496760011         0.750000000
     0.463019997         0.996760011         0.750000000
     0.536979973         0.003239989         0.250000000
     0.872690022         0.856729984         0.250000000
     0.127309978         0.143270016         0.750000000
     0.372690022         0.643270016         0.750000000
     0.627309978         0.356729984         0.250000000
     0.500000000         0.000000000         0.000000000
     0.000000000         0.500000000         0.000000000
     0.500000000         0.000000000         0.500000000
     0.000000000         0.500000000         0.500000000
```

```
CsPbCl3
1.0
        8.2069997787         0.0000000000         0.0000000000
        0.0000000000         8.2550001144         0.0000000000
        0.0000000000         0.0000000000        11.7589998245
   Cl    Cs    Pb
   12     4     4
Direct
     0.776690006         0.223830000         0.038400002
     0.223309994         0.776170015         0.961600006
     0.276690006         0.276170015         0.961600006
     0.723309994         0.723829985         0.038400002
     0.223309994         0.776170015         0.538399994
     0.776690006         0.223830000         0.461600006
     0.723309994         0.723829985         0.461600006
     0.276690006         0.276170015         0.538399994
     0.080380000         0.527079999         0.250000000
     0.919619977         0.472920001         0.750000000
     0.580380023         0.972920001         0.750000000
     0.419620007         0.027079999         0.250000000
     0.987259984         0.958670020         0.250000000
     0.012740016         0.041329980         0.750000000
     0.487259984         0.541329980         0.750000000
     0.512740016         0.458670020         0.250000000
     0.500000000         0.000000000         0.000000000
     0.000000000         0.500000000         0.000000000
     0.500000000         0.000000000         0.500000000
     0.000000000         0.500000000         0.500000000
```

```
CsPbBr3
1.0
        8.2069997787         0.0000000000         0.0000000000
        0.0000000000         8.2550001144         0.0000000000
        0.0000000000         0.0000000000        11.7589998245
   Br   Cs   Pb
   12    4    4
Direct
     0.797219992         0.203759998         0.043269999
     0.202780008         0.796239972         0.956730008
     0.297219992         0.296240002         0.956730008
     0.702780008         0.703760028         0.043269999
     0.202780008         0.796239972         0.543269992
     0.797219992         0.203759998         0.456730008
     0.702780008         0.703760028         0.456730008
     0.297219992         0.296240002         0.543269992
     0.103720002         0.517840028         0.250000000
     0.896279991         0.482159972         0.750000000
     0.603720009         0.982159972         0.750000000
     0.396279991         0.017840028         0.250000000
     0.975910008         0.936670005         0.250000000
     0.024089992         0.063329995         0.750000000
     0.475910008         0.563329995         0.750000000
     0.524089992         0.436670005         0.250000000
     0.500000000         0.000000000         0.000000000
     0.000000000         0.500000000         0.000000000
     0.500000000         0.000000000         0.500000000
     0.000000000         0.500000000         0.500000000
```

```
CsPbI3
1.0
        8.2069997787         0.0000000000         0.0000000000
        0.0000000000         8.2550001144         0.0000000000
        0.0000000000         0.0000000000        11.7589998245
    I   Cs   Pb
   12    4    4
Direct
     0.835449994         0.168099999         0.045189999
     0.164550006         0.831900001         0.954810023
     0.335449994         0.331900001         0.954810023
     0.664550006         0.668099999         0.045189999
     0.164550006         0.831900001         0.545189977
     0.835449994         0.168099999         0.454809994
     0.664550006         0.668099999         0.454809994
     0.335449994         0.331900001         0.545189977
     0.133039996         0.464540005         0.250000000
     0.866959989         0.535459995         0.750000000
     0.633040011         0.035459995         0.750000000
     0.366959989         0.964540005         0.250000000
     0.945100009         0.869149983         0.250000000
     0.054899991         0.130850017         0.750000000
     0.445100009         0.630850017         0.750000000
     0.554899991         0.369149983         0.250000000
     0.500000000         0.000000000         0.000000000
     0.000000000         0.500000000         0.000000000
     0.500000000         0.000000000         0.500000000
     0.000000000         0.500000000         0.500000000
```

Figure S3. The figure illustrates the different bond lengths and bond angles in the four structures

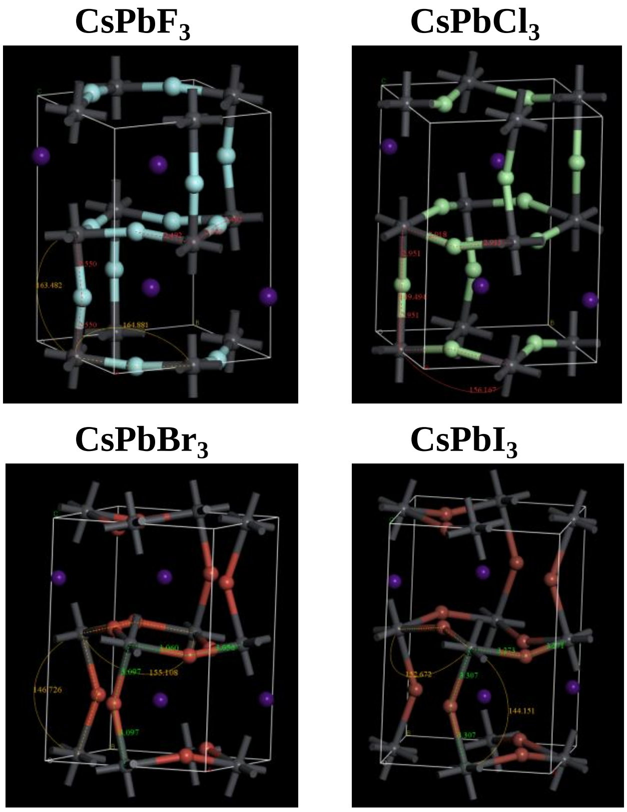

System	Pb-X bond length/ Å		Pb-X-Pb bond angle/ °	
	I	II	I	II
$CsPbF_3$	2.494/ 2.493	2.550	164.758	163.341
$CsPbCl_3$	2.918/ 2.915	2.951	156.167	149.494
$CsPbBr_3$	3.060/ 3.056	3.097	155.108	146.726
$CsPbI_3$	3.273/ 3.271	3.307	152.672	144.151

Figure S4. CASTEP Geometry Optimization and Optimization Convergence

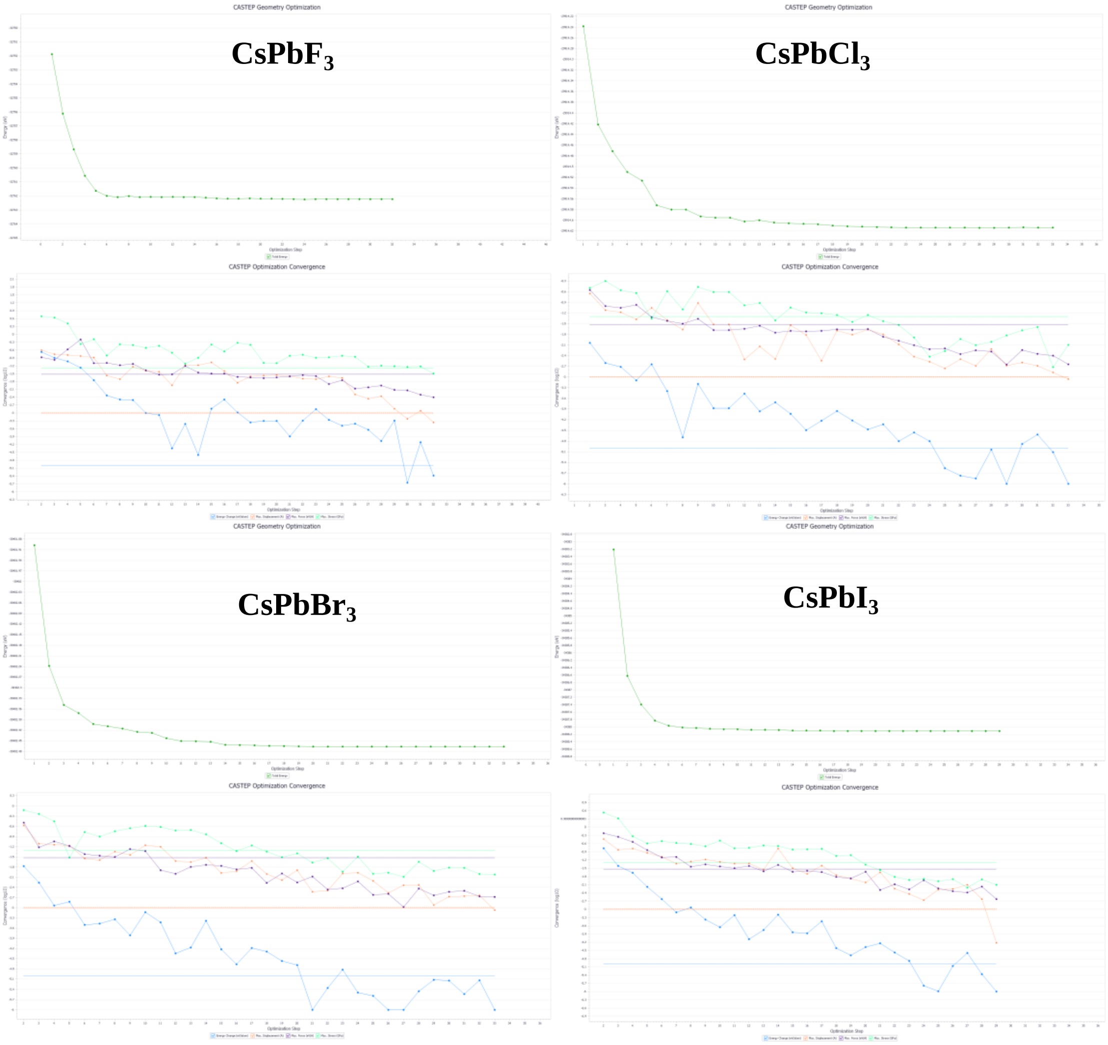

Supplement: Supplementary file 1 [file molecules-28-07643-s001.zip › molecules-2687902-supplementary.pdf]
